# Evaluation of the fate of carcasses and dummies deployed in the nearshore and offshore waters of the northern Gulf of Mexico

**DOI:** 10.1007/s10661-019-7923-0

**Published:** 2020-03-17

**Authors:** Nadia Martin, Veronica W. Varela, F. James Dwyer, Peter Tuttle, R. Glenn Ford, Janet Casey

**Affiliations:** 1 Industrial Economics, Incorporated (IEc), 2067 Massachusetts Avenue, Cambridge, MA 02140 USA; 2 Natural Resource Damage Assessment and Restoration Program, United States Fish and Wildlife Service, Alaska Regional Office, 1011 East Tudor Road, Mail Stop #361, Anchorage, AK 99503 USA; 3 DWH NRDAR Field Office, United States Fish and Wildlife Service, 341 Greeno Road North, Suite A, Fairhope, AL 36532 USA; 4 R.G. Ford Consulting Company, 2735 N.E. Weidler Street, Portland, OR 97232 USA

**Keywords:** Carcass drift, Avian mortality, Deepwater horizon, Oil spill, Gulf of Mexico, Loss at sea

## Abstract

In response to the *Deepwater Horizon* oil spill, federal and state agencies conducted field studies to develop inputs for a shoreline deposition model used to estimate nearshore avian mortality resulting from the spill. A 2011 carcass drift study was designed to generate data on the likelihood that birds that died on the water would deposit along the northern Gulf of Mexico coast (rather than becoming lost at sea). In the case of the *Deepwater Horizon* oil spill, carcass losses at sea accounted for a significant portion of nearshore avian mortality. We evaluate the data collected during the *Deepwater Horizon* carcass drift study and compare the results obtained from the use of avian carcasses versus dummy carcasses (dummies) and the differences between those deployed nearshore versus further offshore. We conclude that, although the use of dummies provided valuable confirmation on the drift patterns of dead birds, dummies drifted greater distances, for longer periods of time, and were more likely to be observed beached compared to avian carcasses, with 64.6% of dummies beaching compared to 17.2% of carcasses. In response to future spills, researchers should account for these potential biases when incorporating dummy drift data into estimates of avian carcass loss. Further, none of the avian carcasses and dummies released more than 40 km from the shoreline made it to shore. In the northern Gulf of Mexico, carcasses that die on the waters farther offshore are unlikely to make it to shore to be captured in a deposition model; therefore, it may be appropriate to utilize a separate methodology to estimate offshore mortality. The applicability of these results to other spill events should be evaluated in the context of the specific spill characteristics.

## Introduction

The number of dead or impaired birds collected or observed along the shoreline after an oil spill or other avian mortality event is often used as an indicator of avian mortality or avian injury. However, the number of birds collected is less than the total number of birds that died because, among other things, (1) shoreline wildlife searchers are unable to find every carcass or debilitated live bird in the areas being searched (referred to as searcher efficiency or detection probability); (2) carcasses do not persist indefinitely on the shoreline in order to be found (carcass persistence); and (3) in the case of aquatic spills/events, not all of the carcasses afloat at sea will drift to the shoreline where they can be found by searchers (carcass loss at sea). Hence, a deposition model is commonly used to estimate the total number of mortalities caused by the event based on the number of birds collected and adjustment factors for detection probability, carcass persistence, and carcass loss at sea (Ford et al. 1987; Page et al. 1990; Piatt et al. 1990; Flint et al. 1999; Ford 2006; IEc 2015a; Amend et al. 2020).

The *Deepwater Horizon* (hereafter the DWH) oil spill began on April 20, 2010, after a mobile drilling unit exploded, caught fire, and eventually sank in the northern Gulf of Mexico. The event resulted in a massive release of oil from the Macondo well, eventually affecting shorelines from Texas to Florida and causing numerous avian mortalities. Following the DWH oil spill, authorized state and federal agencies formed the *Deepwater Horizon* Oil Spill Natural Resource Trustee Council to work together to assess resource injuries, as part of a natural resource damage assessment (NRDA) under the Oil Pollution Act (OPA; 33 U.S.C. §§ 2701–2761). As part of the process to quantify avian injuries, carcasses and live debilitated birds were collected from northern Gulf of Mexico shorelines and field studies were conducted to develop a number of inputs for use in quantifying avian mortality.

The Shoreline Deposition Model (SDM; Amend et al. 2020) was used to estimate the number of birds that likely deposited on the Gulf of Mexico shoreline. The SDM relies on the number of collected birds and adjustment factors for detection probability (Zimmerman and Varela 2020) and carcass persistence (Varela and Zimmerman 2020) to account for birds that were not discovered by shoreline searches. However, the SDM accounts only for those carcasses that deposited on the shoreline. Therefore, an adjustment factor for carcasses lost at sea (that would not have been deposited on the shoreline) was applied to the SDM results (IEc 2015b).

Generally, the loss of avian carcasses at sea is due to either sinking or degradation resulting from natural decomposition or scavenging activity. These two factors can vary depending on the season, currents, and/or the distance a carcass would have to drift before reaching the shoreline (Ford et al. 1987, 1996, 2006; Page et al. 1990). Field studies and models have been used to estimate these factors for spill assessments. Field studies can provide site-specific information, but require time and funding, and may be influenced by differences in environmental conditions at the time of the study compared to the time of the spill impact. Models require inputs and assumptions that can lack site-specificity.

Carcass drift studies have been conducted under a variety of circumstances in order to estimate site-specific beaching probabilities of carcasses drifting at sea, including a number conducted in response to oil spills (Ford et al. 2014a, 1996, IEc 2015b). These studies generally show that a large proportion of the birds that die during an oil spill never beach, but the actual proportion may be highly variable (Hope Jones et al. 1970; Bibby and Lloyd 1977; Bibby 1981; Piatt et al. 1990; Ford et al. 1996). However, with the exception of assessments for the *Nestucca*, *Exxon Valdez*, and *Luckenbach* spills, previous drift studies did not include the tracking of drifting carcasses using radio telemetry. Without the assistance of telemetry, carcasses and dummies may have beached without being recovered, making it difficult to directly compare results to what we observed during the DWH carcass drift study. Further, many of the previous drift studies were conducted at higher latitudes (e.g., California and Alaska; Piatt et al. 1990; Ford et al. 1996) and do not necessarily reflect the relatively warm subtropical conditions in the northern Gulf of Mexico.

Advection models, sometimes used in combination with carcass drift data, can simulate trajectories of floating carcasses (Page et al. 1990; Ford et al. 2001; Carter et al. 2003). Advection models can be helpful for predicting carcass drift if the at-sea distribution of birds is known and the time-specific sinking rates of carcasses can be estimated (e.g., using information on scavenging and carcass loss from previous drift studies conducted in a similar geographic region and time of year). However, estimating the trajectories of floating carcasses as part of an advection model may not be necessary depending on the specific characteristics of the oil spill or the chosen approach to quantifying avian injuries. Piatt and Ford et al. (1996) suggest that conducting a site-specific carcass drift study may be the most accurate way to determine the likelihood of beaching since the recovery rates of marked carcasses in a study would reflect the actual oceanographic conditions and carcass sinking rates of the spill in question.

To improve the accuracy of the estimates of total nearshore avian mortality for the northern Gulf of Mexico, and develop a carcass loss at sea factor specific to the Gulf of Mexico, a carcass drift study was conducted in the summer of 2011. The drift study was designed to provide an estimate of the percentage of carcasses that were likely to reach the shoreline (Ford and Varela 2011). The DWH carcass drift study included the release of both avian carcasses and dummy carcasses (“dummies” designed to float like birds but built using non-degradable materials) at locations near the shoreline and further offshore near the Macondo well site. Data from the study were evaluated to determine the percentage of carcasses likely to have washed ashore in the northern Gulf of Mexico after dying on the water as a result of the DWH spill. Under the assumption that environmental conditions present during the study period (summer 2011) were comparable to those present during the post-spill period for which acute avian mortality was estimated (approximately April–September 2010), the data were used to calculate a Lost at Sea Factor which was applied to the results from the SDM to account for carcass losses at sea.

In this paper, we provide an analysis of the data collected during the 2011 DWH carcass drift study. We conclude that the carcasses and dummies released further offshore have distinct results from those released nearshore. Our analysis includes an evaluation of (1) carcass movement, (2) differences between the fates of carcasses and dummies released nearshore and offshore, and (3) the likelihood that a bird dying in the northern Gulf of Mexico would strand along the northern Gulf of Mexico shoreline. Finally, based on our results, we provide information and recommendations useful for future marine oil spills.

## Methods

The carcass drift study design is described in Ford and Varela (2011) and Ford et al. (2014a). A total of 248 bird carcasses and 66 dummies (together, 314 “drifters”) were outfitted with VHF radio transmitter assemblies (Advanced Telemetry Systems Model Number F1835B) and set adrift in the northern Gulf of Mexico waters between July 16 and August 6, 2011 (Ford et al. 2014a). A total of 200 carcasses and 50 dummies were deployed in the nearshore waters, within 40 km of the coastline off of Louisiana, Mississippi, Alabama, and Florida while 48 carcasses and 16 dummies were released offshore near the Macondo well site. Dummies were used to gain further insight as to how natural processes (e.g., decomposition and scavenging) might impact the probability of a carcass drifting to shore and beaching. Carcasses and dummies (with transmitter assemblies) were tracked using radio telemetry to determine when and where they beached along the shoreline (beach or marsh edges) in the northern Gulf of Mexico. A similar drift study was conducted using dummies as part of the Exxon Valdez oil spill (Ford et al. 1996). Radio-tagged dummies made from styrofoam were deployed as a control in Prince William Sound and the Gulf of Alaska off Kodiak Island in eight separate releases between May 25, 1990 and August 12, 1990.

### Carcass drift study area

The study area included the waters and shorelines of the northern Gulf of Mexico from approximately Atchafalaya, Louisiana to Apalachicola Bay, Florida, including associated coastal marshes and nearshore bayous. This generally coincided with the shoreline area where wildlife searchers surveyed for dead and impaired birds, the tally of which is a primary input to the SDM. Thus, the carcass drift study provides an estimate of the likelihood that carcasses afloat in the nearshore waters and near the Macondo well site would strand along the northern Gulf of Mexico shoreline where searchers conducted surveys for carcasses.

### Drifter assembly

Drifters were designed similarly to Ford et al. (2006). No birds were killed for use in the carcass drift study. Instead, avian carcasses that had been euthanized or collected by various legally permitted programs throughout the USA were salvaged for use in the study. None of the study carcasses were affiliated with oil spills or disease-related mortality events. All carcasses were freshly salvaged, shipped on ice, received fresh (i.e., not frozen), and deployed within 5 days following the death of the bird. Carcasses available for use in this study (*n* = 248) included four gull species: 219 laughing gulls (*Leucophaeus atricilla*), 17 ring-billed gulls (*Larus delawarensis*), 9 herring gulls (*Larus argentatus*), and 3 great black-backed gulls (*Larus marinus*). Approximately 150 species of birds were found during wildlife searches after the DWH spill. Over 45% of the collected birds were gull species and many others were similar in size to gulls; therefore, the gulls used in this study are likely to have behaved similarly to those that died as a result of the spill.

Dummies (*n* = 66) were made out of plastic water bottles sealed inside neoprene sleeves. The dummies were designed to emulate the drift properties of a dead seabird species typical of the northern Gulf of Mexico. However, while similar to avian carcasses in float characteristics, dummies provide a comparison that is not influenced by decomposition and scavenging. Prior to using dummies for this study, a preliminary calibration study was conducted with water bottles and neoprene sleeves to compare float, sink, and drift characteristics of the dummies to seabird carcasses with masses of approximately 500 to 800 g. It was determined that a 650-g dummy best mimicked those of actual bird carcasses (Ford et al. 2014a).

**Fig. 1:**
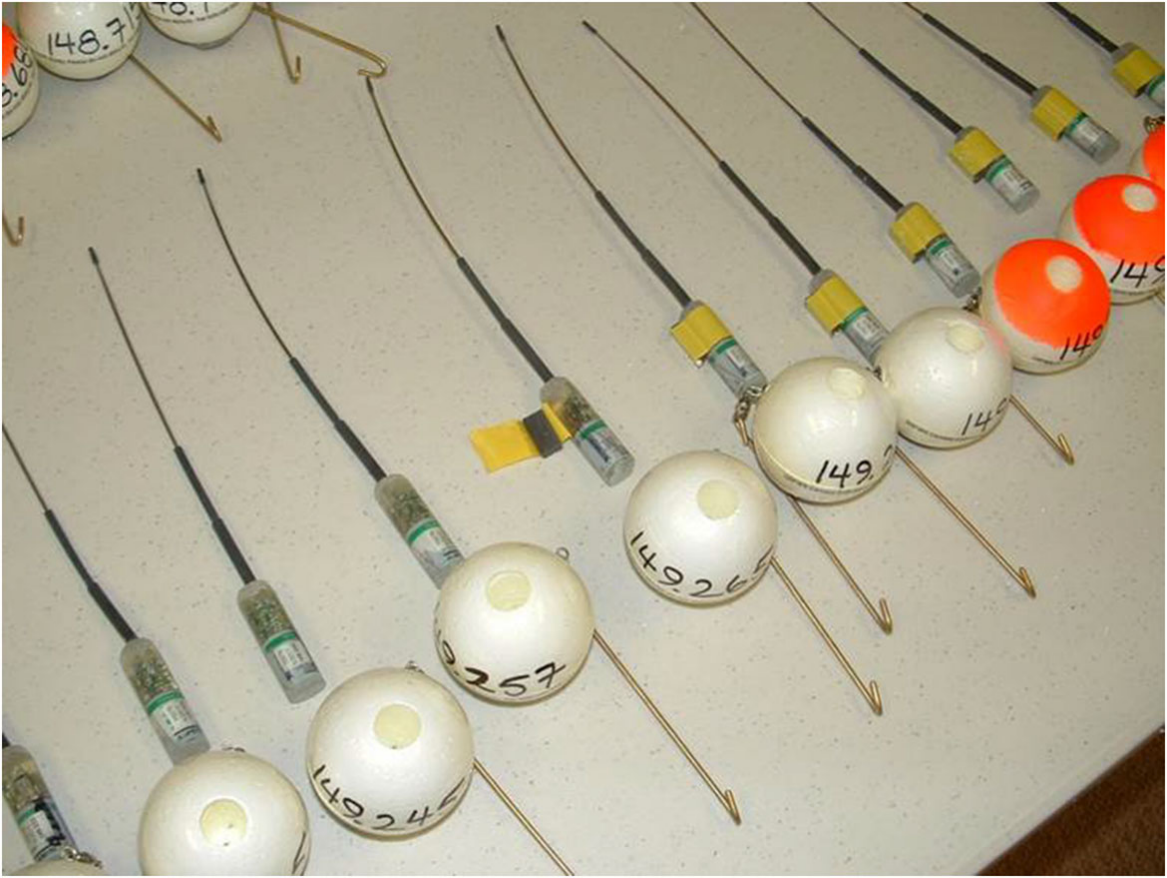
Photograph of transmitters and floats used in the 2011 DWH oil spill carcass drift study

VHF radio transmitters were used for both carcasses and dummies. The transmitters were approximately 83 g, resin-encased and originally designed by the manufacturer to be implanted in fish. Transmitters were crystal controlled three-stage models that provided an enhanced range. The expected lifespan of the lithium batteries ranged between 185 and 366 days. Transmitters were encased within closed-cell polystyrene spheres that were weighted on the bottom to ensure that the radio antenna was protruding upwards (Fig. 1). Stainless steel wire was used to attach the transmitter to both carcasses and dummies. During drifter deployments, transmitter signals were verified as operational immediately before and after each drifter was released into the water.

### Drifter deployment and tracking

**Fig. 2:**
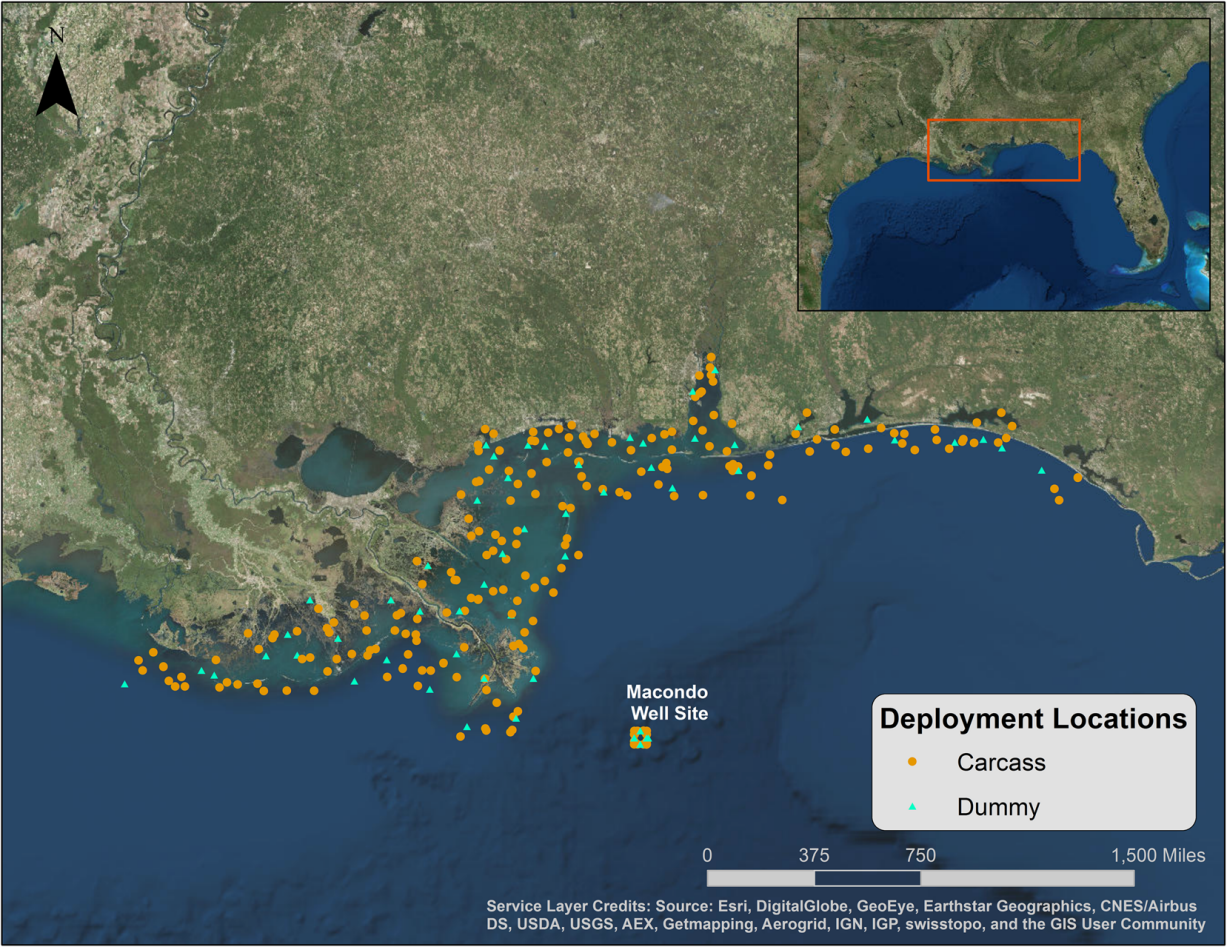
Deployment locations of carcasses and dummies relative to the shoreline and Macondo well site location. Carcass deployment locations are illustrated using a circle and dummy locations using a triangle

Drifters were deployed over a range of dates from July 16 to August 6, 2011 (Ford et al. 2014a) to capture as many variations in environmental conditions as possible during the study period. All carcasses and dummies were deployed directly into the water and tracked for at least 14 days after their release date (August 20, 2011) unless a drifter was retrieved onshore by study personnel or until the radio signals ceased (Ford et al. 2014a). Previous studies suggest that most carcasses should cease to float (i.e., sink or strand) between 14 and 21 days (Wiese 2003; Ford et al. 1987; Hope Jones et al. 1970). These previous studies were conducted in cold water environments. We expected carcasses deployed in the warmer environment of the Gulf of Mexico to decompose and sink faster than those deployed in cold water and so assumed a tracking period of at least 14 days would be sufficient. When the radio signal could no longer be detected, we assumed that the carcass had either sunk, been scavenged, floated out of the study area (i.e., outside of the northern Gulf of Mexico), or the transmitter had failed. The drifters released nearshore were deployed at random locations within the areas with the highest bird densities as determined from low-level aerial surveys conducted as part of a different natural resource damage assessment during the summer of 2010 (Ford et al. 2014b). Half of the carcasses and dummies released at the well site (24 and 8, respectively) were released by a boat, and the remainder released by a helicopter (Fig. 2).

Throughout the northern Gulf of Mexico, drifters were tracked by crews in planes, boats, and on foot, with crews regularly flying thousands of miles each day during the study period to search for radio signals. When a signal was located by the aerial crew, tracking personnel would record the position where the signal strength was strongest or box the transmitter location by flying a series of concentric diminishing rectangles around the transmitter. When radio signals indicated that a carcass or dummy might have washed ashore, the aerial tracking crew contacted boat or foot tracking crews to search for the drifter. Once a ground crew established that the drifter had beached, the crew photographed it and recorded the date, location, habitat type where it was observed beached, and the condition (if the drifter was a carcass). The condition of the carcass was recorded using one of nine carcass condition codes: “Int.” = intact, equivalent to no scavenging; “Dist.” = disturbed, skin broken, mostly intact; “PR” = pectorals removed but organs present; “OR” = organs removed but pectorals present; “SB” = skin and bones; “PG” = fragmentary, pelvic girdle only remaining; “Wing” = fragmentary, one or both wings only remaining; “Skin” = feathers attached to skin fragments remaining; and “Miss” = missing, float and transmitter only, no part of carcass remains, equivalent to removed.

### Data processing and analysis

Each drifter was assigned to one of the three categories: (1) lost at sea, (2) beached, or (3) excluded from analysis. We considered whether the carcass or dummy had been observed after being beached along the shoreline, the location where it was confirmed as beached, and the condition when it was observed beached. We evaluated the data collected during deployment and radio telemetry tracking of each drifter to determine the number of carcasses and dummies that beached and were observed by field teams. After being released, drifters generally succumbed to one of the three fates: (1) they were never detected again, (2) they were detected by air and/or ground crews but could not be directly observed or recovered, or (3) they beached and were located by ground crews.

We compared the results of carcasses to dummies, including the percentage that beached, the number of days drifting prior to beaching, and the relationship between beaching and the distance from the shore where a drifter was released (hereafter referred to as deployment distance). To investigate the potential relationship between beaching and deployment distance, we used the GPS locations for the deployment combined with aerial imagery of the shoreline from 2011. Deployment distance was calculated as the linear distance from the release location to the closest shoreline, including breakwaters and small islets. We evaluated the statistical significance for various comparisons using an alpha value of 0.05.

#### Drifters excluded

A carcass or dummy was excluded from the analysis when there was incomplete information to evaluate whether it had beached along the shoreline. Carcasses were excluded from analysis when information was not available to confirm the location and/or condition of the carcass on the day of first beaching. These situations included when citizens not involved in the study tampered with a study carcass and when a carcass suspected as beached was located in an area not accessible to study personnel (either due to access restrictions or safety precautions). Dummies were excluded from analysis when there was incomplete information regarding when and where a dummy beached due to area access restrictions for ground crews.

#### Drifters lost at sea

Carcasses and dummies categorized as “lost at sea” included drifters that were never observed beached (including those never detected after being deployed and those that were detected but never observed beached), transmitters that were found beached with the carcass missing, or carcasses that only made it to shore with the assistance of the float and transmitter (as determined by the authors). Some carcass drifters were found beached in such an advanced state of decomposition that we determined the carcass should have sunk but for the assistance of the float and transmitter.

#### Drifters beached

Carcasses and dummies categorized as “beached” include those drifters that made it to the shoreline and, for carcasses, only those that could be verified as being in a condition that would have allowed the carcass to reach the shore without the assistance of the float and transmitter. We defined the date of beaching as the first day that a ground crew directly observed a drifter on the shoreline. Some carcasses were left in the field and revisited for a few days after the first beached observation. For dummies, we identified the first date the dummy was observed beached by identifying the first field encounter by ground crews where the crew recorded a shoreline habitat (emergent marsh, sandy beach, and rip rap were considered shoreline habitats). Aerial crews may not have detected a drifter immediately after it beached, but we assumed that the carcass or dummy likely had stranded on the shoreline within 24 h of when the field team confirmed it as beached.

**Fig. 3:**
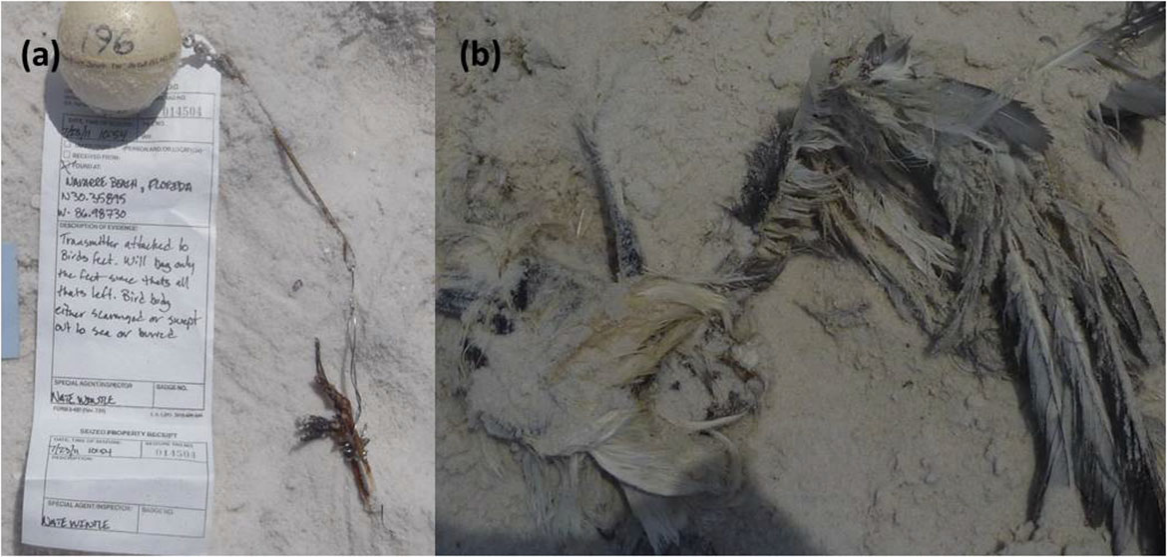
**a** Photograph of one carcass classified as the leg bones only remaining (LBO) scavenging condition; **b** photograph of one carcass classified as the skin and bones (SB) scavenging condition

To determine whether a carcass beached in a condition that likely would have made it to shore in the absence of the float and transmitter, we considered data recorded by ground crews, including photographic data and radio-tracking information. We verified all recorded carcass conditions by reviewing photographs of the carcasses. We made changes to the carcass condition determination based on photographs, particularly for those carcasses categorized as skin and bones. We identified carcasses that included numerous bones and feathers (including wing feathers) that would make it to shore without the assistance of a float and transmitter and those including leg bones only (Fig. 3) which would not (IEc 2015b). Carcasses considered to have been capable of beaching without float and transmitter assistance were in conditions ranging from intact to something more than leg bones attached to a pelvis (Int., Dist., PR, OR, and SB). Carcasses consisting of only leg bones (with or without a pelvis) or wing bones to which the transmitter float was wired (PG, Wing, Skin, LBO, and Miss.) were considered likely to have only made it to shore due to the buoyancy of the transmitter float. If the condition of the carcass was unknown on the first day it was recorded beached, we used all available information including subsequent days’ photographs or notes to make a determination. We also evaluated the time series of data to verify when a carcass was beached versus still afloat near a shoreline. We used the location of the drifter, the habitat type, and the type of field crew to confirm (i.e., ground or boat) when a carcass or dummy had beached and to verify the date it beached.

## Results

### Nearshore

Of the 200 carcasses and 50 dummies deployed in the nearshore waters (within 40 km of the shoreline), 14 carcasses and 2 dummies were excluded from the analysis because their fates could not be verified (e.g., ground crews could not reach them due to access restrictions and could not confirm their condition or that they had beached) (Table 1). A total of 154 carcasses and 17 dummies were considered lost at sea, either because they were never detected again after deployment, were detected by ground crews after they were released but never directly observed beached, or they were detected and observed beached along the shoreline but considered lost at sea due to their conditions (i.e., they were in a condition that we determined was unlikely to have beached without the assistance of the transmitter float) (Table 1). The remaining 32 carcasses were considered to have beached in a condition that would have likely beached without the assistance of the transmitter float and a total of 31 dummies were considered beached and were retrieved by field crews (Table 1).

**Table 1:** Fate of carcasses and dummies deployed nearshore

Fate	# of carcasses	# of dummies
Excluded	14 (7%)	2 (4%)
Lost at sea	154 (82.8%)	17 (35.4%)
Never detected after deployment	18 (9%)	4 (8%)
Detected after deployment, but never observed beached	80 (40%)	13 (26%)
Detected and observed beached along the shoreline, but considered “lost at sea”	56 (17.2%)	0 (0%)
Beached	32 (17.2%)	31 (64.6%)
Detected and observed beached along the shoreline, and considered “beached”	32 (17.2%)	31 (64.6%)
Total	200	50

The mean (M) deployment distances (km) among the nearshore drifters that beached was significantly different between dummies (*M* = 5.63, standard deviation (*SD*) = 6.54) and carcasses (*M* = 2.55, *SD* = 3.85; two-sample, two-tailed *t* test, *t*(48) = 2.27, *p* = 0.03). Among the drifters released in the nearshore environment that beached, the mean number of days to beaching differed significantly between dummies (*M* = 9.00, *SD* = 7.43) and carcasses (*M* = 4.66, *SD* = 4.08; two-sample, two-tailed *t* test, *t*(46) = 2.87, *p* < 0.01). However, for both dummies and carcasses, each analyzed separately, there was no evidence of a relationship between deployment distance and the number of days to beaching (linear regression *R*^2^ = 0.08 and < 0.01, respectively). Additionally, for nearshore carcasses, the mean deployment distances were significantly different between those that beached (*M* = 2.55, *SD* = 3.85) and those that were lost at sea (*M* = 7.37, *SD* = 6.84; two-sample, two-tailed *t* test, *t*(78) = 5.51, *p* < 0.001). However, for dummies released in the nearshore environment, the mean deployment distances were not significantly different between those that beached (*M* = 5.63, *SD* = 6.54) and those that were lost at sea (*M* = 7.72, *SD* = 6.66; two-sample, two-tailed *t* test, *t*(46) = 1.05, *p* = 0.30).

On average, carcasses and dummies that were observed beached drifted approximately 22 km from the point of release to the beaching location (measured as the straight line distance between the point of release and the beaching location). On average, dummies drifted farther (average of 33.7 km) before beaching than did carcasses (average of 10.0 km). The distance dummies drifted ranged from 0.5 to 161 km and the distance carcasses drifted ranged from 0.3 to 41 km. The farthest traveling drifter (that was observed beached in the study area) was a dummy that was deployed offshore of the Mississippi barrier islands and drifted eastward before beaching along Pensacola Beach in Florida (161 km). Taking into account all of the transmitter signal fixes documented for each carcass and dummy, the total drifting distance for each was greater than the straight line distance between the release location and beaching location. Within the nearshore waters, there was no clear pattern to the direction the carcasses and dummies drifted with some drifting north and eastward and others drifting westward.

### Offshore

For the carcasses (*n* = 48) and dummies (*n* = 16) that were deployed near the well, 100% were considered lost at sea because they were never detected again after deployment or were detected by crews but not observed beached; none were ever observed beached within the study period (Table 2).

**Table 2:** Carcasses and dummies deployed offshore considered beached or lost at sea

	# of carcasses	# of dummies
Lost at sea	48 (100%)	16 (100%)
Never detected after deployment	20 (42%)	1 (6%)
Detected after deployment, but never observed beached	28 (58%)	15 (94%)
Beached	0 (0%)	0 (0%)
Total	48	16

## Discussion

### Nearshore and offshore carcasses

Based on our evaluation of the DWH carcass drift study data, we calculated the percentage of birds that died in nearshore waters that were likely to make it to the shoreline and used this value to develop a Lost at Sea Factor to apply to the SDM results. For nearshore carcasses, on average, those that stranded were released closer to the shore than those that were lost at sea. This suggests that carcasses (but not dummies) deployed farther from shore, but within the nearshore waters, may have been more likely to be affected by scavengers or decomposition.

When deployed offshore near the Macondo well site, none of the carcasses or dummies beached in the study area during the study period, likely due to many drifting out of the study area. Since birds dying far offshore during the summer in the northern Gulf of Mexico were very unlikely to beach, these offshore birds would not have been included in nearshore avian injury quantification estimates calculated using the SDM (IEc 2015a, b). This finding allowed for a separate approach to estimate offshore avian mortality, similar to the approach used for the T/V Puerto Rican oil spill (PRBO 1985; IEc 2015c). Recognizing that nearshore and offshore avian injuries are distinct ensured that there was no double-counting (i.e., counting the same individual birds in both approaches). A similar approach should be considered for future spills if similar drift results are observed.

### Carcass and dummy movements in the northern Gulf of Mexico

There are a number of factors that influence the drift pattern of carcasses and dummies. The temperature of the water at sea can influence the fate of drifting avian carcasses, since carcasses in warmer waters may be scavenged more quickly (Himes Boor and Ford 2016). One of the most prominent factors is local winds and currents. The DWH carcass drift study was conducted in 2011, a year after the DWH oil spill and it is possible that overall wind and current patterns differed between 2011 and 2010. We did not statistically compare the winds and currents between 2010 and 2011.

However, our observation that there was no discernible drift pattern in the nearshore environment is supported by the overall northern Gulf of Mexico current patterns described by Johnson (2008). Johnson synthesized 23 years of northern Gulf of Mexico temperature and current data to describe generalized monthly and seasonal currents. At any location and/or time, these temperatures and currents may vary from the generalized pattern. The DWH carcass drift study was conducted during July and August which corresponds with the summer seasonal pattern (June–August) described by Johnson. Our purpose in evaluating Johnson’s data was not to predict where drifters might be found for our study but rather to evaluate if our findings were in general agreement with the patterns identified over a 23-year period. This was important since the drifter study was conducted the year following the spill (2011) but applied to the year the spill occurred (2010).

**Fig. 4:**
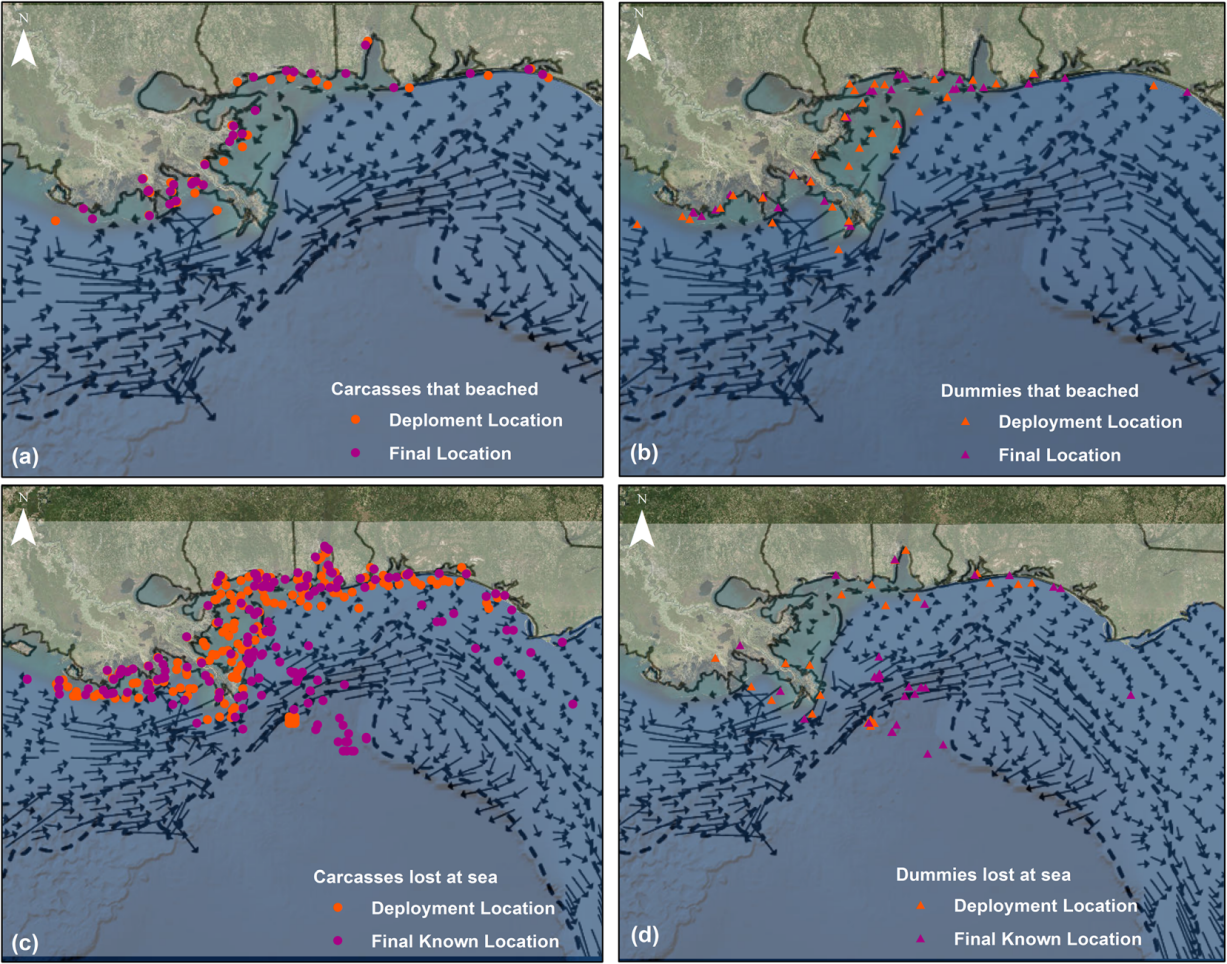
Carcass and dummy deployment and final locations in relation to Gulf of Mexico currents in the summer. The water currents (Johnson 2008) are illustrated using arrows on the figure; **a** includes deployment and final locations for carcasses that beached; **b** includes deployment and final locations for dummies that beached; **c** includes deployment and final known locations for carcasses lost at sea; and **d** includes deployment and final known locations for dummies lost at sea. Baselayer credits: Esri, DigitalGlobe, GeoEye, Earthstar Geographics, CNES/Airbus DS, USDA, USGS, AEX, Getmapping, Aerogrid, IGN, IGP, swisstopo, and the GIS User Community

During the summer, seasonal winds toward the north are most prevalent. However, Johnson describes that tides, interactions with deep-basin events, and buoyancy outflows from river and estuarine areas all contribute to the nearshore currents. This mix of factors provides a very complex nearshore current pattern which supports our observation of no clear overall drift direction for carcasses or dummies deployed nearshore (Fig. 4). However, toward the edge of the continental shelf, a summer pattern of west to east currents emerges (Johnson 2008). According to Johnson (2008), the predominant currents along the continental slope of the northern Gulf of Mexico are generally toward the east during the summer seasonal pattern. Moving eastward along the Gulf of Mexico shoreline where the continental shelf’s edge curves southward, the seasonal current (Fig. 4) also flows southward toward the large-scale currents of the Loop Current and its spin-off eddies. Carcasses and dummies deployed near the Macondo well site, which is on the continental slope, likely became caught in these currents, inhibiting the movement of drifters toward northern Gulf of Mexico shorelines and may explain why we did not find any well site deployed drifters during the study. Although these large-scale currents are predominantly deep-ocean phenomena, they can interact with currents on the continental shelf (Johnson 2008). After termination of the carcass drift study, 68 days after its deployment in nearshore waters off the Chandeleur Islands in Louisiana, a carcass drifter (with only leg bones attached) was discovered on the east coast, in Fort Lauderdale, Florida. A second drifter deployed southeast of Grand Isle was also found on the east coast of Florida, over 3 months after its deployment. This indicates some birds that died nearshore can become entrained in large-scale currents and drift far from the spill leading eventually to the Florida Current that exits in the Gulf of Mexico between Florida and Cuba.

### Drift distances and time to beaching

We expected a relationship between deployment distance and time to beaching, such that the drifters released closer to shore would beach sooner. However, we found no such relationship for drifters that beached (and were released within the nearshore waters). This may be associated with nearshore winds and currents within our study area that may have been locally highly variable, keeping some carcasses adrift longer than others.

**Fig. 5:**
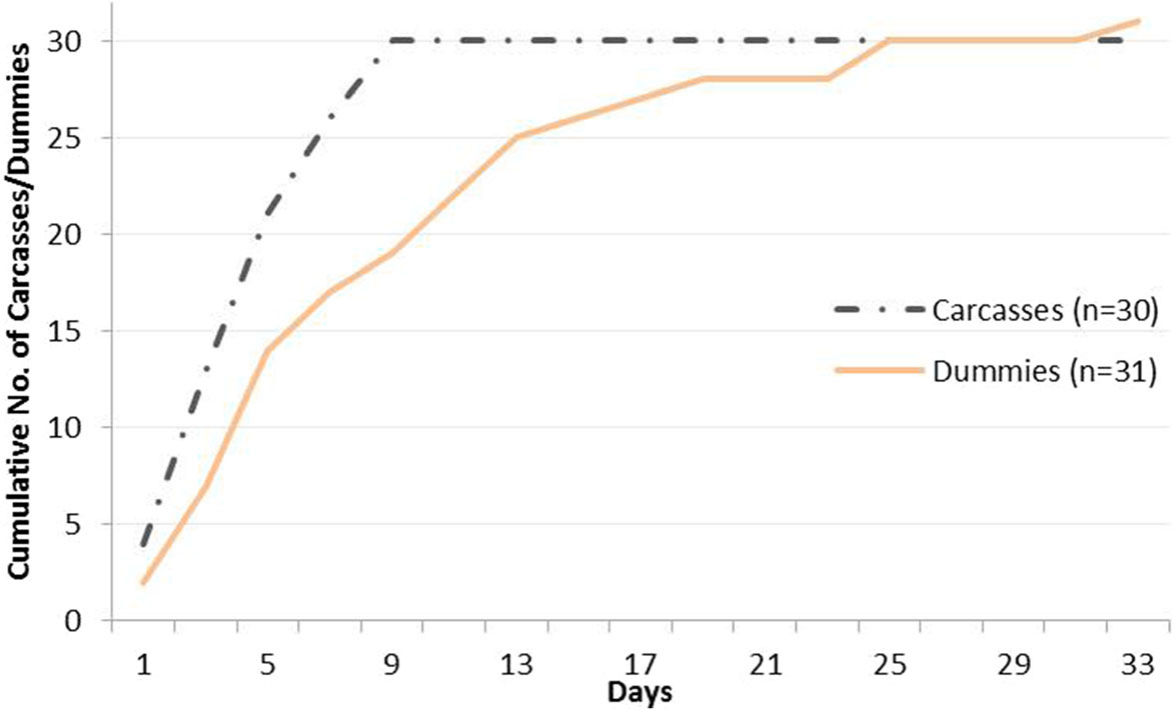
Cumulative number of carcasses and dummies that reached the shore by day

On average, it took longer for dummies to beach within the study area than carcasses. It took an average of 5 days between deployment and beaching for carcasses to beach. Of the carcasses that beached, 94% did so within 9 days and 66% within 4 days. For dummies, it took an average of 9 days to beach. Of the dummies that beached, 90% did so within 18 days and 70% within 11 days (Fig. 5). The maximum number of days between deployment and beaching for carcasses that beached was 20 days, whereas the maximum for dummies was 32 days. Of those carcasses that were considered beached only with the transmitter’s assistance, 73% did so within 10 days (with a range between 1 day and 22 days).

### Difference in beaching between carcasses and dummies deployed nearshore

For carcasses released nearshore, those that stranded along the shoreline tended to have been released closer to shore than those that were lost at sea. This suggests that carcasses released in the nearshore environment (but not dummies) deployed farther from shore may have been more likely to be affected by scavengers and decomposition and thus disappeared at sea or may have been entrained in currents that would have prevented the carcass from beaching.

The fate of drifters that did not beach is uncertain but is likely due to a variety of reasons including scavenging, decomposition, and drifting out of the study area. Although transmitter signals were verified during the release of each drifter, transmitter failure cannot be ruled out as another possible reason for carcasses or dummies being considered lost at sea. Carcasses, which are subject to decomposition and scavenging, were lost at sea more often than dummies. For drifters deployed in the nearshore waters, 64.6% of the dummies were observed beached and retrieved, compared to 17.2% of carcasses. Since less than one-fifth of the carcasses made it to shore, this suggests a fairly high level of decomposition and scavenging of avian carcasses at sea in the northern Gulf of Mexico. Additionally, while we attempted to adjust for float and transmitter effects by reviewing the photographs of each carcass to evaluate the carcass condition and likelihood of it beaching without the assistance of the transmitter float, it is possible that we underestimated the influence of the transmitter float. In that case, even fewer birds would have actually made it to shore but for the assistance of the floats and the 17.2% beaching probability could represent an upper bound.

For carcasses that made it to shore within three or fewer days of deployment (*n* = 13), 77% were categorized as intact or mostly intact, but skin broken or partly disturbed. Of those that drifted for four or more days before beaching (*n* = 19), only 11% were categorized as mostly intact, and the rest were degraded or scavenged to skin and bones, fragmentary, or organs removed. The loss rate of dummies was significantly lower than the loss rate for carcasses, indicating that decomposition and other biological processes likely played an important role in carcass disappearance. However, we cannot rule out the possibility of some dummies being lost at sea due to scavenging. Some carcasses and dummies also may have drifted out of the study area. All of those deployed far offshore, near the site of the well, never made it to the shoreline. The loss of all dummies and carcasses from the well site indicates that birds dying far offshore are unlikely to have drifted to the shoreline for discovery by search crews. Further, since all of the dummies (which are not as likely to be subject to scavenging and are not subject to decomposition), as well as the carcasses, deployed further than 40 km from the shore, were lost, many of these drifters are likely to have drifted out of the study area.

### Comparison to other drift studies

Previous estimates of oil spill-related avian mortality have accounted for at-sea losses of carcasses, which have varied from as low as 41% to as high as 99% (Piatt and Ford 1996). In the case of DWH, carcass losses at sea accounted for a significant portion of nearshore avian mortality (IEc 2015b). Himes Boor and Ford (2016) also analyzed the data collected during the DWH carcass drift study using a mark-recapture model to derive the beaching probability of dead birds afloat at sea. The authors found that avian carcasses at sea had a range of 11 to 16% chance of beaching. Their estimate is similar to our 17.2% of nearshore avian carcasses beaching. The difference is likely due to the definition of “beaching.” For example, Himes Boor and Ford considered carcasses that reached the shore outside of their defined study area (e.g., farther west or inland) as lost rather than beached. Our approach considered only whether a carcass reached the shore in a condition that would have allowed the carcass to beach without the assistance of the float and transmitter.

## Conclusions

In summary, the DWH carcass drift study provided valuable information that was critical for developing the Lost at Sea Factor used to estimate the number of birds killed in the nearshore environment as a result of the spill (IEc 2015a, b). We found that offshore drifters were very unlikely to make it to the shoreline, which led to a separate estimate of offshore avian mortality. Nearshore carcass losses at sea accounted for a significant portion of the nearshore avian mortality estimate (IEc 2015a). Only 17.2% of avian carcasses deployed in the nearshore environment drifted to a beach or marsh edge in a condition that would have likely stranded without the assistance of the transmitter and/or float compared to 64.6% of dummies released nearshore. However, these results may be very different from carcasses drifting in colder environments (Himes Boor and Ford 2016). The applicability of these results to other spill events should be evaluated in the context of the specific spill characteristics.

While great effort was taken to ensure that dummies were similar in size and floated similarly to bird carcasses, the results show that the dummies drifted greater distances, for longer periods of time, and were more likely to be observed beached on the shoreline compared to carcasses. This observation indicates the need to recognize potential inaccuracies that could be introduced by using data related to dummies rather than carcasses in drift studies. To avoid underestimating carcass loss at sea, we recommend estimating carcass drift using bird carcasses rather than dummies or adjusting calculations for the potential bias in dummy results.

## References

[CR1] Amend, M., Martin, N., Berger, M., Dwyer, F. J., Donlan, M., & Varela, V. (2020). Avian injury quantification using the shoreline deposition model and model sensitivities. *Environmental Monitoring and Assessment,*10.1007/s10661-019-7922-1. 10.1007/s10661-019-7922-1 PMC7078175 32185519

[CR2] Bibby CJ (1981). An experiment on the recovery of dead birds from the North Sea. Ornis Scandinavica.

[CR3] Bibby CJ, Lloyd CS (1977). Experiments to determine the fate of dead birds at sea. Biological Conservation.

[CR4] Carter HR, Lee VA, Page GW, Parker MW, Ford RG (2003). The 1986 *Apex Houston* oil spill in central California: seabird injury assessments and litigation process. Marine Ornithology.

[CR5] Flint PL, Fowler AC, Rockwell RF (1999). Modeling bird mortality associated with the *M/V/Citrus* oil spill off St. Paul Island, Alaska. Ecological Modeling.

[CR6] Ford RG (2006). Using beached bird monitoring data for seabird damage assessment: the importance of search interval. Marine Ornithology.

[CR7] Ford, R. G., & Varela, V. (2011). Using radio telemetry to determine the fates of bird carcasses drifting in the northern Gulf of Mexico (bird study #1D), final – 12 July, 2011. *Deepwater Horizon Report*. https://pub-dwhdatadiver.orr.noaa.gov/dwh-ar-documents/787/DWH-AR0033993.pdf. Accessed 28 Mar 2016.

[CR8] Ford, R. G., Page, G., & Carter, H. (1987). Estimating mortality of seabirds from oil spills. *Oil Spill Conference, 1987*, 547–551.

[CR9] Ford RG, Bonnell ML, Varoujean DH, Page GW, Carter HR, Sharp BE, Heinemann D, Casey JL (1996). Total direct mortality of seabirds from *Exxon Valdez* oil spill. American Fisheries Society Symposium.

[CR10] Ford, R. G., Himes Boor, G. K., & Ward, J. C. (2001). Seabird mortality resulting from the *M/V New Carissa* oil spill incident, February and March 1999. Prepared for U.S. Fish and Wildlife Service, Oregon Fish and Wildlife Office. May.

[CR11] Ford, R. G., Strom, N. A. & Casey, J. L. (2006). Acute seabird mortality resulting from the *S. S. Luckenbach* and associated mystery oil spills, 1990-2003. Prepared for California Department of Fish and Game Office of spill prevention and response. April.

[CR12] Ford, R. G., Casey, J. L., Strom, N. A., & Williams, W. A. (2014a). Draft final end of study data report, using radio telemetry to determine the fates of bird carcasses drifting in the northern Gulf of Mexico (carcass drift study – Bird study #1D). *Deepwater Horizon Technical Report*. https://pub-dwhdatadiver.orr.noaa.gov/dwh-ar-documents/788/DWH-AR0065410.pdf. Accessed 1 Mar 2016.

[CR13] Ford, R. G., Davis, J. N., Briggs, K. T., Strom, N., Casey, J. L., & Williams, W. A. (2014b). Draft report avian injury quantification: live oiled bird model - abundance and distribution. *Deepwater Horizon Technical Report*. https://pub-dwhdatadiver.orr.noaa.gov/dwh-ar-documents/790/DWH-AR0096611.pdf. Accessed 28 Mar 2016.

[CR14] Himes Boor, G. K., & Ford, R. G. (2016) Beaching probability estimate for adrift seabird carcasses resulting from the Deepwater Horizon Oil Spill. *In review*.

[CR15] Hope Jones P, Howells G, Rees EIS, Wilson J (1970). Effect of ‘Hamilton Trader’ oil on birds in the Irish Sea in May 1969. British Birds.

[CR16] Industrial Economics, Incorporated (IEc). (2015a). *Deepwater Horizon*/Mississippi Canyon 252 Oil Spill Natural Resource Damage Assessment Technical Report: quantification of nearshore avian mortality using the shoreline deposition model and lost at sea factor. September 3, 2015. https://pub-dwhdatadiver.orr.noaa.gov/dwh-ar-documents/788/DWH-AR0302137.pdf. Accessed 1 Mar 2016.

[CR17] Industrial Economics, Incorporated (IEc). (2015b). *Deepwater Horizon*/Mississippi Canyon 252 Oil Spill Natural Resource Damage Assessment Technical Report: estimating the proportion of bird carcasses lost at sea after DWH oiling mortality. August 31, 2015. https://pub-dwhdatadiver.orr.noaa.gov/dwh-ar-documents/788/DWH-AR0152635.pdf. Accessed 1 Mar 2016.

[CR18] Industrial Economics, Incorporated (IEc). (2015c). *Deepwater Horizon*/Mississippi Canyon 252 Oil Spill Natural Resource Damage Assessment Technical Report: estimating the offshore mortality of birds killed by DWH oil. August 31, 2015. https://pub-dwhdatadiver.orr.noaa.gov/dwh-ar-documents/788/DWH-AR0011784.pdf. Accessed 1 Mar 2016.

[CR19] Johnson DR (2008). Ocean surface current climatology in the northern Gulf of Mexico. Funded by the marine fisheries initiative program of NMFS/NOAA.

[CR20] Page GW, Carter HR, Ford RG (1990). Numbers of seabirds killed or debilitated in the 1986 Apex Houston Oil Spill in Central California. Studies in Avian Biology.

[CR21] Piatt JF, Ford RG (1996). How many seabirds were killed by the Exxon Valdez Oil Spill?. American Fisheries Society Symposium.

[CR22] Piatt JF, Lensink CJ, Butler W, Kendziorek M, Nysewander DR (1990). Immediate impact of the *Exxon Valdez* oil spill on marine birds. Auk.

[CR23] Point Reyes Bird Observatory (PRBO). (1985). The impacts of the T/V Puerto Rican oil spill on marine birds and mammal populations in the Gulf of the Farallones, 6–9 November 1984. *Special scientific report, prepared with assistance from international bird rescue*. March.

[CR24] Varela, V. & Zimmerman, G. (2020). Persistence of avian carcasses on sandy beaches and marsh edges in the northern Gulf of Mexico. *Environmental Monitoring and Assessment, *10.1007/s10661-019-7920-3. 10.1007/s10661-019-7920-3 PMC7078173 32185585

[CR25] Wiese FK (2003). Sinking rates of dead birds: improving estimates of seabird mortality due to oiling. Marine Ornithology.

[CR26] Zimmerman, G., & Varela, V. (2020). Detection probabilities of bird carcasses along sandy beaches and marsh edges in the northern Gulf of Mexico. *Environmental Monitoring and Assessment, *10.1007/s10661-019-7924-z. 10.1007/s10661-019-7924-z PMC7078141 32185513

